# Adolescent deliveries in semi-urban Cameroon: prevalence and adverse neonatal outcomes

**DOI:** 10.1186/s13104-017-2555-3

**Published:** 2017-06-26

**Authors:** Tsi Njim, Valirie Ndip Agbor

**Affiliations:** 1 Centre for Global Health and Tropical Medicine, Nuffield Department of Medicine, University of Oxford, Oxfordshire, UK; 2 Health and Human Development Research Group (2HD), Douala, Cameroon; 3 Ibal Sub-Divisional Hospital, Oku, North West Region Cameroon

**Keywords:** Adolescent deliveries, Adverse neonatal outcomes, Cameroon

## Abstract

**Objectives:**

Adolescent pregnancies are high risk due to the increased probability of adverse outcomes; as adolescents are usually considered to be ill-equipped to deal with the burden of pregnancy. We sought to determine the prevalence of adolescent deliveries in a secondary-level care hospital in semi-urban Cameroon-Bamenda, the adverse neonatal outcomes and to assess if previous obstetric history could preclude adolescents from having adverse outcomes in their present pregnancy.

**Results:**

The prevalence of adolescent deliveries was 8.7% (95% CI 7.01–10.73%). The neonates of adolescent mothers were more likely to have severe asphyxia (OR 4.0; 95% CI 1.2–12.9; p = 0.03) and low birth weight (OR 2.4; 95% CI 1.3–4.4; p < 0.01). The neonates of primipara adolescents were just as likely to have complications as multipara adolescents. The prevalence of adolescent deliveries (8.7%) in the Regional Hospital Bamenda is high. Their babies are at a high risk of adverse neonatal outcomes irrespective of their previous obstetric history (previous delivery) emphasising that adolescents are generally ill-prepared to deal with pregnancy. Strategies to reduce the prevalence of adolescent deliveries should be investigated and implemented in view of attaining the sustainable development goals.

## Introduction

Adolescent deliveries are considered to be high risk pregnancies due to the potential increased risk of adverse outcomes to both the mother and the baby [1]. Adolescent girls are usually considered to be physiologically ill-prepared to handle the physical, social and mental changes associated with pregnancy [2]. The reduction of the prevalence of adolescent pregnancies in developing countries is therefore a public health imperative on the road to the attainment of the sustainable development goals by 2030 [3]. This is essential as adolescent pregnancies indirectly act as a hindrance to the attainment of these goals. Pregnant adolescents are: likely to be out of school with an increased risk of socioeconomic hardship and poverty (Goal 1, 2 and 4) and to die from pregnancy-related complications (Goal 3) [4].

In developing countries like Cameroon, the burden due to adolescent pregnancies is high with recent studies showing a prevalence range of 9.3–9.9% in urban and suburban areas [2, 5]. In some regions of the country like the North, early marriages are usually promoted as part of the culture [2] and it is popular belief that adolescents who are married have more favourable outcomes than their single counterparts. It was however shown recently in a study that the marital status of adolescents did not reduce their predisposition to adverse outcomes [2]. It will therefore be important to investigate if multipara adolescents have more favourable outcomes than primipara adolescents—another cultural belief held in these regions.

In this report, we sought to determine the prevalence of adolescent deliveries in a semi-urban area in the country—Bamenda; its adverse neonatal outcomes and whether being a multiparous adolescent precluded them from having poor neonatal outcomes. The results should help policy-makers in drafting adolescent-friendly health programmes and will assist in portraying a more complete picture of the epidemiology and burden of this problem.

The data used for analysis in this note comes from part of a larger study to assess the burden of low birth weights in neonatal care units in the RHB.

## Main text

### Study setting and participants

The data was collected from the maternity of the Regional Hospital Bamenda (RHB) in the Northwest region of Cameroon from November 2015–January 2016. Bamenda is a suburban town which is the capital of the Northwest region of Cameroon (one of the ten regions in the country). It has an estimated population of 500,000 inhabitants. The BRH has a total bed capacity of about 600 beds and serves as main referral hospital in the region, as well as teaching hospital for the Faculty of Health Sciences, University of Bamenda. The maternity of this hospital conducts more deliveries than other health facilities in the region. It is run by five obstetricians/gynaecologists.

All consenting women with singleton pregnancies were recruited at delivery during the study period. Women with abortions (gestational age <28 weeks) were excluded.

### Study procedure

Ethical approval was obtained from the Ethical committee of the RHB and administrative approval from the Regional Delegation of Public Health for the Northwest region. Women at the point of delivery during the study period were monitored from labour till delivery and the following information was obtained: Socio-demographic characteristics (age, sex and marital status); obstetric history (gravidity and parity) and antenatal care history for the current pregnancy. The length of the labour was noted and the mode of delivery recorded (normal vaginal delivery vs. caesarean section). After delivery, the neonates were assessed for viability (breathing, beating of the heart, pulsation of the umbilical cord or definite movements of voluntary muscles), and their APGAR scores at the 1st min were determined. The baby’s weight was measured to the nearest gram. After delivery, the mother was approached, the study explained to her and only those who provided written informed consent were included in the study. This was done only after delivery to reduce coercion due to labour pains. Participants were monitored until the day of discharge. A total of 886 participants were recruited for the study.

### Data management and statistical analysis

The data were secured in a confidential and private location. Participants were referred to by identification numbers and the consent forms were kept separate from the questionnaires. Both could only be linked by a coding sheet available only to the investigators. Data were analysed using Epi Info version 7.2.0.1. Means (standard deviations) were used to summarise continuous variables and proportions for categorical variables.

Firstly, the proportion of adolescents (defined as age <19 years) who had complications (neonatal asphyxia, neonatal death, low birth weight, preterm deliveries and caesarean sections) were compared to their adult counterparts using Fisher’s exact tests. Statistical significance was set at p < 0.05. The adolescents were then divided into primiparas (those having their first delivery) and multiparas (those having their second or above delivery) and the proportions of the complications were compared using the Fisher’s exact test.

### Results

The mean age of the study participants was 26.6 ± 5.3 years with a mean parity of 2.3 ± 1.3. Over half (51.7%) of the neonates born were males. The mean age for delivery for adolescents was 18.0 ± 1.1 while that for their adult counterparts was 27.4 ± 4.8. The prevalence of adolescent deliveries over the study period was 8.7% (77/886) (95% CI 7.0–10.7%). Also, 11.6% of adolescents were multiparas compared to 73.9% of their adult counterparts (Table 1). The overall rate of caesarean sections during the study period was 12.5%.

**Table 1:** Sociodemographic characteristics of study participants in the Bamenda Regional Hospital from November 2015–January 2016

Characteristic	Adolescents		Adults	
	Frequency	Proportion	Frequency	Proportion
Marital status				
Married	5	6.5	533	65.9
Single	72	93.5	276	34.1
Parity				
Primiparas	61	88.4	184	26.1
Multiparas	8	11.6	521	73.9
Preterm				
Yes	6	7.8	42	5.2
No	71	92.2	767	94.8
Gender of baby				
Male	41	54	413	51.5
Female	35	46	389	48.5

Adolescents were more likely to have: neonates with severe asphyxia (APGAR <4) (OR 4.0; 95% CI 1.2–12.9; p = 0.03) and neonates with low birth weight (LBW) (weight ≤ 2600 g [6]) (OR 2.4; 95% CI 1.3–4.4; p < 0.01) (Table 2). There was no difference in the proportions of adolescents who had caesarean sections, high birth weight (weight ≥ 3850 g [7]), preterm deliveries and neonates who died when compared with their adult counterparts (Table 2).

**Table 2:** Neonatal outcomes of adolescent deliveries in the Bamenda Regional Hospital from November 2015–January 2016 amongst 886 singleton deliveries

Outcomes	Adolescents		Adults		OR	95% CI	p value
	Frequency	(%)	Frequency	(%)			
Asphyxia (Apgar <7)	6	7.9	33	4.1	2.0	0.8–5.0	0.1
Severe asphyxia (Apgar <3)	4	5.3	11	1.4	4.0	1.3–12.9	0.03
Neonatal mortality	3	4.0	11	1.4	3.0	0.8–10.9	0.1
Low birth weight	14	18.2	70	8.7	2.4	1.3–4.4	<0.01
High birth weight	4	5.2	89	11	0.4	0.2–1.2	0.07
Caesarean section	13	16.9	96	11.9	0.7	0.4–1.2	0.2
Preterm delivery	6	7.8	42	5.2	1.5	0.6–3.8	0.2

Adolescents who were multiparas were just as likely to have neonates with severe asphyxia (3.3% vs. 12.5%, p = 0.3) and LBW (19.7% vs. 0%, p = 0.2) as those who were primiparas (Table 3).

**Table 3:** Neonatal outcomes of primipara adolescent deliveries compared with multipara adolescent deliveries in the Bamenda Regional Hospital from November 2015–January 2016

Outcomes	Primiparas		Multiparas		OR	95% CI	p value
	Frequency	(%)	Frequency	(%)			
Asphyxia (Apgar <7)	4	6.7	2	25.0	4.7	0.7–31.0	0.1
Severe asphyxia (Apgar <3)	1	12.5	2	3.3	4.1	0.3–51.8	0.3
Low birth weight	0	0	12	19.7	–	–	0.2
High birth weight	3	12.5	1	4.9	0.4	0.03–4.0	0.4
Caesarean sections	10	16.4	3	37.5	3.8	0.7–19.8	0.09

### Discussion

In this report, we determined that the prevalence of adolescent deliveries in the RHB was 8.7%. Neonates of adolescent mothers were more likely to have LBW and severe neonatal asphyxia. The neonates of adolescent primiparas were just as likely to have adverse outcomes as the neonates of adolescent multiparas.

The prevalence of adolescent deliveries in the RHB (8.7%) fell within the range of prevalence in the national territory (6.87–26.51%) [1]. It was however lower than the prevalence of 9.9% obtained in Buea a similar semi-urban setting in the country [2]. This prevalence is also higher than that in developed countries. In Sweden for example, the prevalence of adolescent deliveries decreased from 7.7% in the 1970s to 1.6% in 2003 [8]. Meanwhile, the prevalence in the Netherlands is as low as 4% [9]. The high prevalence in these settings could be explained by the poor level of sexual education in most of the regions in the country [2]. This warrants the need for more comprehensive sexual education programmes and the investigation of strategies to reduce the prevalence of adolescent deliveries to reduce the associated disease burden.

Neonates born to adolescent mothers were more likely to have severe neonatal asphyxia and LBW. This was consistent with results from other studies carried out in a semi-urban setting in the country [2], urban settings in Cameroon [5, 10] and in other sub-Saharan African countries [11]. Adolescents due to lack of proper reproductive education generally have poor antenatal care attendance [2]. The association of poor antenatal care attendance and LBW has been well established [6]. Also, low birth weight neonates are quite likely to suffer from birth asphyxia [6]. This could explain the association of adolescent deliveries with LBW and asphyxia as observed in this study. This highlights the need for specialist attention for the neonates of adolescent mothers and the need for appropriate resuscitative techniques and platforms considering the high morbidity associated with LBW and asphyxia like hypoxic ischaemic encephalopathy. Also, the need to scale-up ANC services in sub-Saharan African countries is apparent [12].

There was no significant difference in mortality amongst neonates born to adolescents or adults. This was consistent with findings from a study in the Southwest region of the country where adolescents were just as likely to have stillbirths as adults [2]. Neonates from adolescent mothers were just as likely to be born at term as neonates from their adult counterparts. This finding is contrary to that observed by several authors [2, 10, 11] and warrants further investigation.

Neonates born to adolescents who were multiparas were as likely to have adverse outcomes as those born to primiparas. It is generally believed that adolescents who are married or have an obstetric history of a previous delivery have more favourable outcomes than their single and primiparous counterparts. A similar study in a semi-urban area of Cameroon has already shown that neonates of married adolescents were just as likely to have complications as neonates of single adolescents [2]. This shows that adolescents are generally physiologically ill-prepared to cope with the burden of pregnancy irrespective of their social or previous obstetric history. These pregnancies are therefore at a higher risk of complications and warrant preventive strategies to curb its prevalence and associated morbidity. However, due to the small number of multiparous adolescents in the study, these results need to be confirmed by larger studies in the country.

### Conclusions

Adolescent deliveries pose a huge problem in semi-urban settings in Cameroon with one in ten deliveries occurring amongst adolescents. Their babies are at a high risk of adverse outcomes irrespective of their previous obstetric history. Strategies to reduce the prevalence of adolescent deliveries and its associated complications need to be investigated and implemented in the country.

## Limitations

The study was carried out in one health facility and cannot be used for generalisations for the rest of the country. This study is however necessary to build the cartography of the adolescent deliveries situation in Cameroon to aid in policy-making and clinical decisions. Also, the prevalence of adolescent deliveries obtained from this study underestimates the burden of adolescent pregnancies in Cameroon as several girls may have had abortions, excluding them from the study or may have given birth at home and could therefore not be recruited into the study.

## Abbreviations

RHB: Regional Hospital Bamenda
OR: odds ratio
CI: confidence interval
LBW: low birth weight
